# Benchmarking of hospital information systems: Monitoring of discharge letters and scheduling can reveal heterogeneities and time trends

**DOI:** 10.1186/1472-6947-8-15

**Published:** 2008-04-19

**Authors:** Martin Dugas, Markus Eckholt, Holger Bunzemeier

**Affiliations:** 1 Department of Medical Informatics and Biomathematics, University of Münster, Domagkstr. 9, D-48149 Münster, Germany; 2 IT center, University Hospital of Münster, Germany; 3 Business division medical management, University Hospital of Münster, Germany

## Abstract

**Background:**

Monitoring of hospital information system (HIS) usage can provide insights into best practices within a hospital and help to assess time trends. In terms of effort and cost of benchmarking, figures derived automatically from the routine HIS system are preferable to manual methods like surveys, in particular for repeated analysis.

**Methods:**

Due to relevance for quality management and efficient resource utilization we focused on time-to-completion of discharge letters (assessed by CT-plots) and usage of patient scheduling. We analyzed these parameters monthly during one year at a major university hospital in Germany.

**Results:**

We found several distinct patterns of discharge letter documentation indicating a large heterogeneity of HIS usage between different specialties (completeness 51 – 99%, delays 0 – 90 days). Overall usage of scheduling increased during the observation period by 62%, but again showed a considerable variation between departments.

**Conclusion:**

Regular monitoring of HIS key figures can contribute to a continuous HIS improvement process.

## Background

The Joint Commission [1], which is responsible for accreditation of more than 15,000 health care organizations in the United States, defines benchmarking as: "Continuous measurement of a process, product, or service compared to those of the toughest competitor, to those considered industry leaders, or to similar activities in the organization in order to find and implement ways to improve it".

Benchmarking plays an important role for healthcare quality information systems [2]. More specific, benchmarking of hospital information systems (HIS) is a relatively new area of research, that will provide methods for healthcare quality information management and clinical outcomes research.

An important issue in benchmarking is definition of suitable performance indicators. However, publications regarding HIS performance indicators that can be determined automatically from routine HIS are very sparse at present. For HIS benchmarking, these key figures should be good measures of HIS function and at the same time available in the routine HIS. Overall hospital performance measures like length of stay have limited value for HIS benchmarking, because HIS is only one of many factors (like availability of qualified medical personnel) with influence on this indicator. To analyze HIS-function regularly, an automated approach would be beneficial.

Time-to-completion of discharge letters and usage of patient scheduling are relevant for quality management and efficient resource utilization in a hospital; therefore we developed and applied benchmarking to these specific aspects of HIS.

We focused on the following objectives:

1. How can completeness and timeliness of discharge letters be measured in an automated way for a commercial HIS?

2. Are there specific patterns of documentation for certain medical specialties?

3. How can usage of scheduling be measured and analyzed in an automated way for a commercial HIS?

## Methods

The overall goal was to identify best practices and weaknesses regarding documentation and scheduling within departments. Therefore all key figures are stratified by department. More specific, we aimed to assess efficiency of improvement measures related to HIS function and to observe time-trends.

We implemented performance indicators for the University hospital of Münster, Germany, a tertiary care referral center with approximately 1500 beds, using the report generator of ORBIS^® ^from Agfa Healthcare [3]. Currently, ORBIS^® ^is applied to following HIS functions: clinical documentation, administrative documentation, order-entry and scheduling. Statistical analysis was performed with R [4].

To assess general technical performance of the HIS, we measured time to switch user accounts including user interactions manually with a stopwatch. Due to heterogeneity of hardware and network components, we defined several workstations at different locations within the hospital (high, typical, average performance) and calculated average time values. Measurements were carried out monthly on workday mornings which correspond to typical workload of the system.

We combined performance indicators from our billing system (inpatients per day) with figures from clinical documentation (discharge letters per day) to estimate completeness of HIS documentation: While almost every patient is registered into the billing system, how many discharge letters are stored in the HIS?

From a quality management perspective, timeliness of discharge letters is particularly relevant for patient-follow-up. Therefore we analyzed time to completion as well as completeness of discharge letters by department. Completeness was defined according to availability of electronically signed discharge letters in the central IT system. Using the report generator of our HIS, we produced lists of case numbers for a specified time interval of discharge, stratified by department. Cases of accompanying persons, who are very common in pediatrics, were eliminated, because documentation is only required for patients. With a second report we identified discharge letters for these episodes of care and calculated the time span between patient discharge and completion of documentation. We generated plots of the empirical cumulative distribution function of completeness by number of days after patient discharge.

Independent of discharge letter analysis, we studied usage of the electronic patient scheduling system. We determined average number of patient appointments per day by department. This analysis was carried out each month during one year. We chose one month as observation period for practical reasons, because there is a monthly review meeting of the IT center to discuss these results. We decided to report average appointments per day instead of appointments per month, because absolute values are smaller and better comprehensible (e.g. 40 per day versus 1200 per month).

We discussed these performance indicators regularly in an internal review meeting of the IT center. If there were indications of organizational issues within departments related to HIS function – i.e. low HIS performance in a specific department without evidence for technical problems – benchmarking figures were presented and discussed with the management of the respective specialty.

## Results

We generated benchmarking reports once per month for one year (from July 2006 to June 2007). According to the billing system, 207 +/- 15 patients (mean +/- standard deviation) were admitted per day. This parameter was relatively stable and we did not observe a significant time trend. Time to switch user accounts was 10.5 +/- 1.9 s (mean +/- standard deviation), which is an indicator of technical performance. During the observation period there was no evidence for a trend regarding this parameter.

### Completeness and timeliness of discharge letters

The number of discharge letters per day per department (averaged over one month) ranged from 0 to 36. Some departments use specialized subsystems for documentation without connection to the central system, which explains zero values. A couple of departments use to some extent standard text processing without centralized storage of documentation. This can lead to low completeness of documentation within HIS.

**Figure 1:**
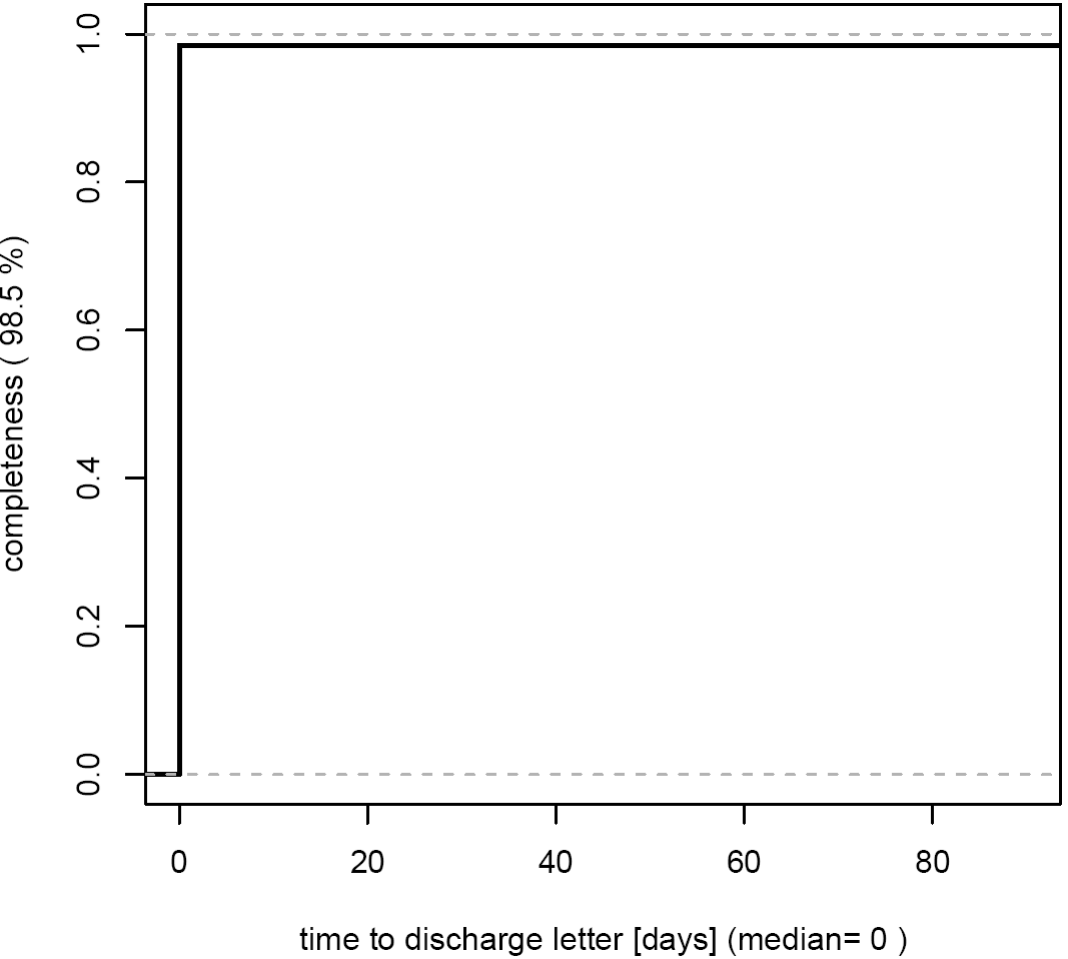
**Completeness by timeliness of documentation (CT-plot).** This department provides highly complete discharge letters (completeness 98.5%) and no delays – best case. 201 cases from March 2007 were analyzed.

**Figure 2:**
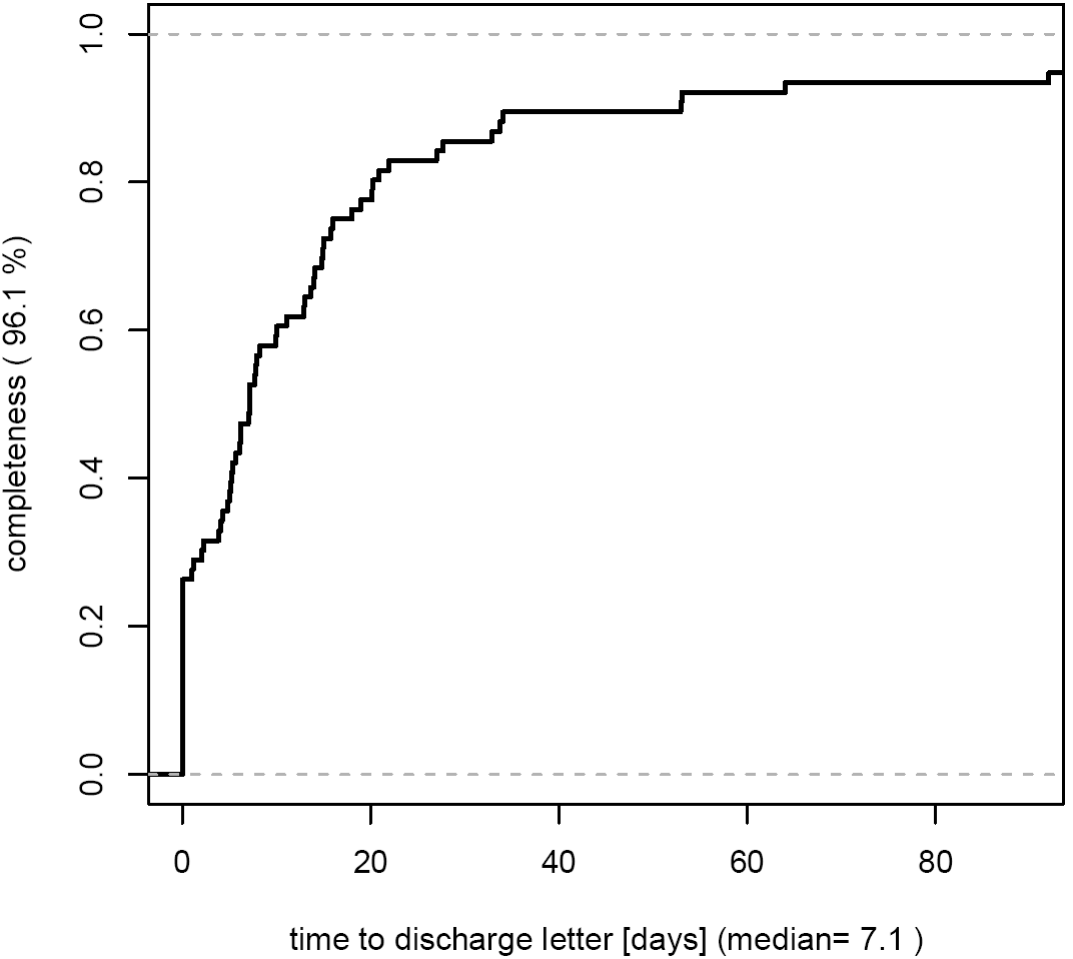
**This specialty reaches a relatively high level of HIS discharge letter completeness (96.1%), but there are relevant delays in documentation (median time span from patient discharge to letter 7 days).** 76 cases from March 2007 were analyzed.

**Figure 3:**
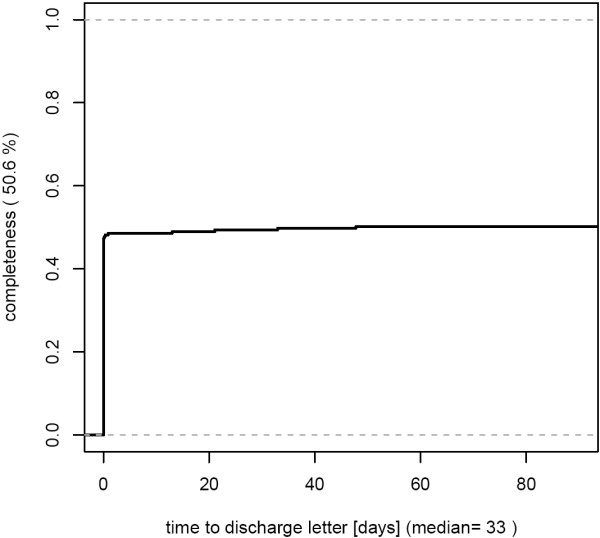
**This department shows low completeness of discharge letters (50.6%), but good results with respect to timeliness. **245 cases from March 2007 were analyzed.

**Figure 4:**
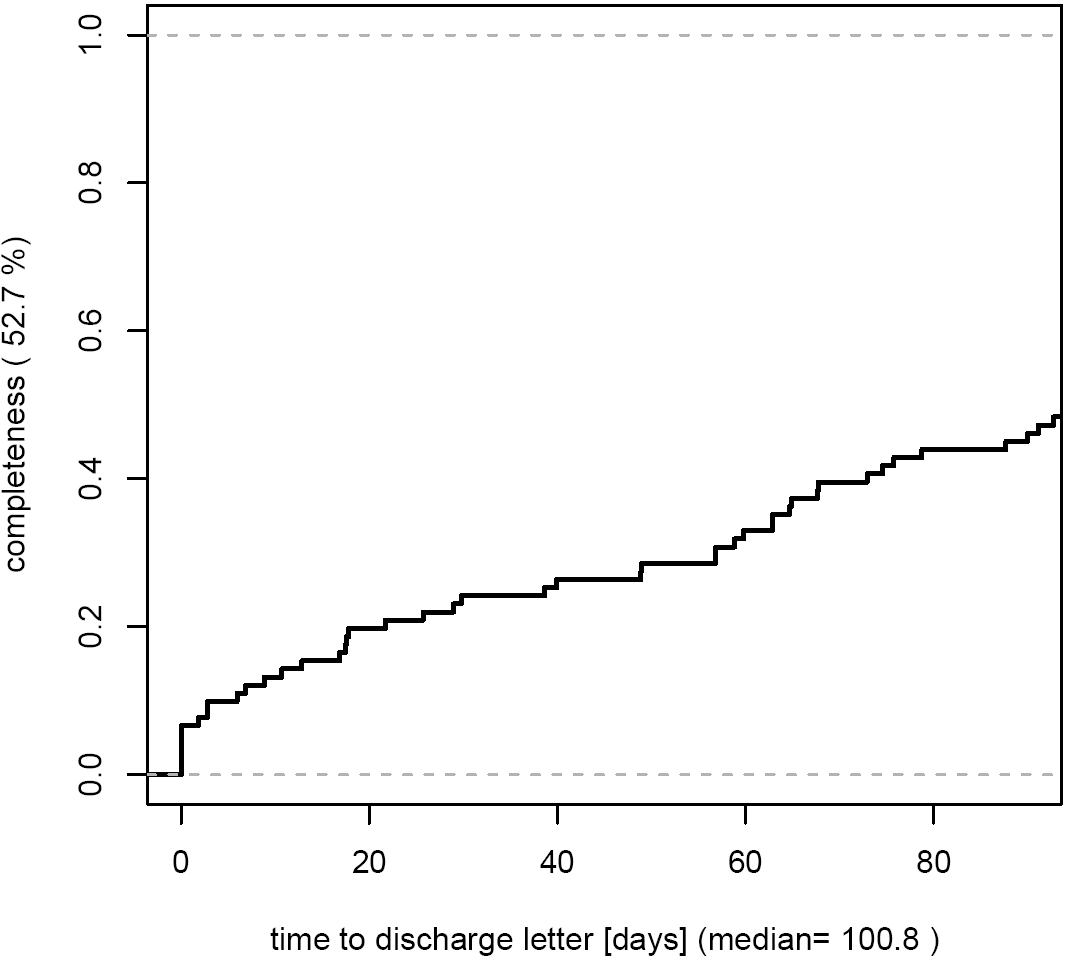
**Combination of incomplete and delayed documentation (completeness 52.7%).** 91 cases from March 2007 were analyzed.

We plotted completeness by timeliness of documentation (CT-plot), specifically completeness by time to discharge letter. The following documentation patterns were observed:

- best case: high completeness of letters without delays [Figure 1]

- high completeness, but relevant delays of documentation [Figure 2]

- low completeness without delays [Figure 3]

- worst case: low completeness combined with delayed documentation [Figure 4]

### Scheduling

**Figure 5:**
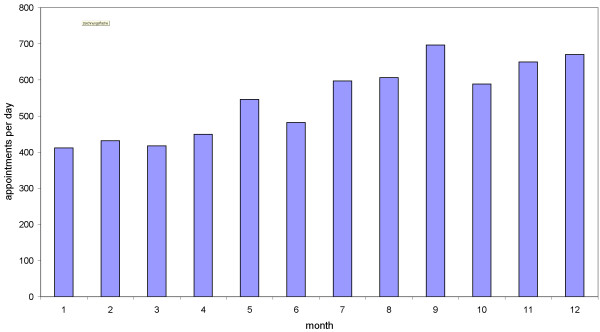
**Overall number of electronic scheduling appointments per day (y-axis) by month (x-axis).** During the observation period scheduling was introduced in several departments.

**Figure 6:**
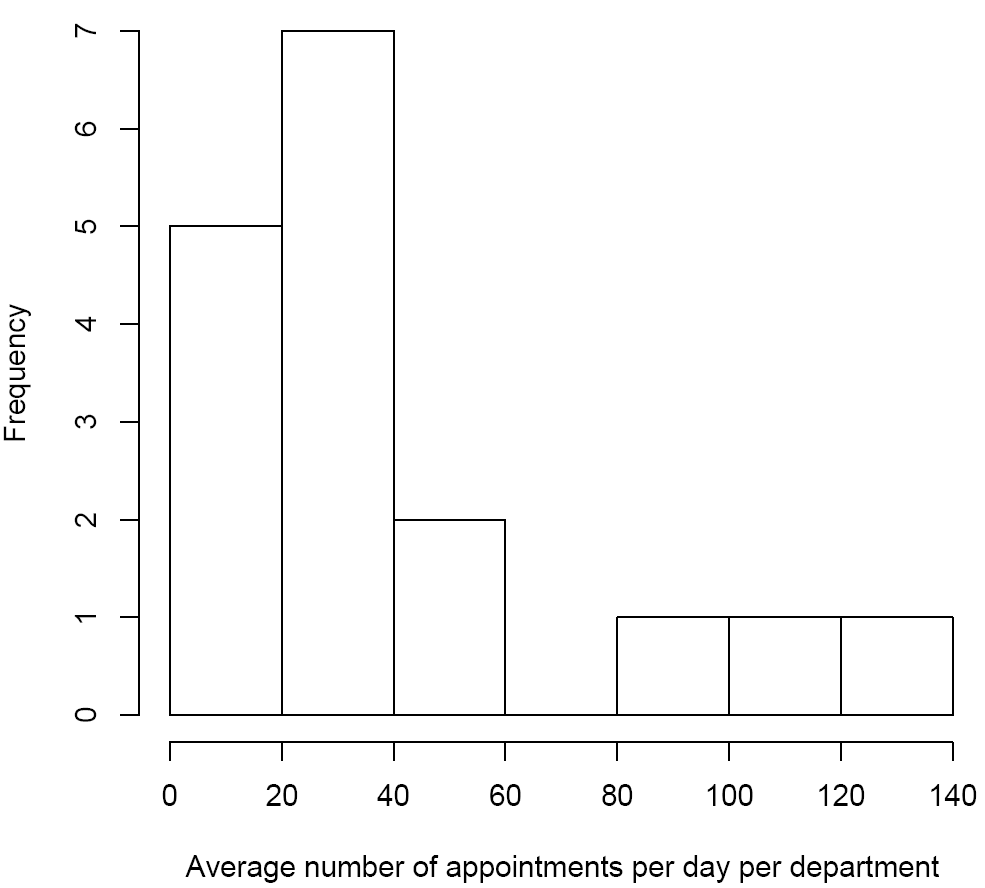
**Histogram of number of appointments per day per department (analysis for month 12).** Seven departments manage on average between 20 and 40 appointments per day electronically. Usage of electronic scheduling varies between less than 20 and more than 120 appointments per day.

During the observation period, electronic patient scheduling was expanded in several departments of the hospital. [Figure 5] presents the time trend of scheduling appointments, starting from 412 per day [month 1], raising to 670 [month 12] per day (62% increase). [Figure 6] shows the frequency distribution of electronic scheduling by department for month 12. Five departments manage on average up to 20 patient appointments per day electronically, three departments 80 or more. These results indicate a relevant variation concerning computer-based management of appointments. This might be explained by differences in number of patients per day between departments. "Big departments" – in terms of number of cases – are expected to manage more appointments than "small" departments. Interestingly, there are small departments with many electronic appointments and vice versa (data not shown).

## Discussion

Our analysis demonstrates that there can be substantial heterogeneity of HIS usage within the same hospital with identical HIS and IT infrastructure. Because our HIS was implemented more than five years ago, this cannot be attributed to novelty of the system.

First, we analyzed technical infrastructure – general computer function, time to switch users, time to search patients etc. – to exclude technical failures as reasons for this heterogeneity.

We discussed results with key users from several departments and identified in the majority of cases organizational issues – like endorsement by senior management, training activities – and different needs of different specialties as reasons for these heterogeneities. In particular, we observed a substantial variation both with respect to completeness and timeliness of discharge letters between departments.

Though, analysis of completeness of discharge letters is affected by different use of specialized subsystems or standard word processing without centralized data management. Specifically the latter form of documentation is problematic, because access to discharge letters is impeded and audit requirements are not satisfied. In two departments we provided specific HIS user training for secretary personnel and observed a significant improvement of completeness in the HIS.

Usage of HIS changes over time, especially when new function modules are being introduced. HIS performance figures can help to objectively assess the progress of HIS projects. This information can be used to steer project resources – for instance, to provide additional user training. In addition, the sustainability of HIS workflows can be audited – to what extent is the HIS really being used in routine care?

HIS benchmarking can be applied to identify best practices: Reviews with key users from departments with good and bad performance indicators can be performed to elucidate options for improvement activities. In our case we identified a wide variation regarding HIS scheduling: Some departments had on average more than 100 electronic patient appointments per day while others had less than 5 even though absolute patient numbers were in the same order of magnitude. In our case, these results motivated several departments to extend electronic patient scheduling.

HIS monitoring activities are relevant for quality management. For instance, timely completion of discharge letters is relevant for continuity of patient care. Regular monitoring is required to verify quality objectives like 95% of physician letters should be completed within one week after patient discharge. In our case, some departments generated a relevant proportion of discharge letters with general purpose text processing and did not store them into the HIS, which is problematic for data security reasons and leads to incomplete electronic patient records. This problem can be identified and addressed by our benchmarking approach. By regular analysis, time-trends and adoption of process changes can be observed and assessed.

Systematic information management in hospitals demands for a strategic information management plan [5]. Performance metrics can measure to what extent objectives are achieved. There are also commercial services for HIS benchmarking (e.g. KLAS vendor rating [6]). However, publications regarding HIS performance indicators that can be determined automatically from routine HIS are very sparse at present. Müller [7] presents an HIS monitoring system embedded in a strategic information management infrastructure. She determined number of documents per case and also identified the issue of local text processing. In our approach, we focused on discharge letters and also addressed the aspect of timeliness. Further developments in medical documentation standards, in particular HL7 clinical document architecture (CDA) [8] with its clinical document code, will facilitate methods for HIS benchmarking.

Monitoring of HIS usage addresses only one, but an important aspect of evaluation: to what extent is the system actually being used in routine operation? Particularly in the health care setting it is known that adoption of an IT system can be problematic [9] – installing license keys and offering some user training does not warrant success. In comparison with various types of surveys, performance metrics are limited in scope, but can be automated and are therefore more cost-efficient in the long run.

So far, benchmarking in health care systems focused mainly on medical outcome and economical issues, i.e. length of stay benchmarking [10]. HIS benchmarking adds further information to the management process of a hospital. Clearly, there is a need for standardized HIS performance figures to enable external benchmarking between hospitals with different HIS. Internal benchmarking is easier to accomplish, because there are less restrictions regarding data access and exchange within one organization. If other hospitals adopt and refine our approach, a consensus process to define generally accepted HIS performance figures could be started. In particular, standard HIS metrics would be beneficial to assess HIS cost-benefit ratio in a more objective manner.

Our approach is focused on very few basic HIS functionalities. It could be extended to address important subsystems like laboratory information management systems (LIMS) or radiology information systems (RIS) as well as enterprise resource planning (ERP). Given the heterogeneity between specialties, similar departments should be compared with each other. Definition of similarity and selection of appropriate HIS performance figures certainly will be a matter of debate, because many metrics may be influenced by factors outside the HIS – like patient characteristics, quality of the management and motivation of staff.

## Conclusion

From our experience it is worthwhile to proceed with HIS benchmarking, in particular regarding discharge letters and scheduling. It can reveal heterogeneities and time trends and thereby contributes to a continuous improvement process which is also beneficial for the patients.

## Abbreviations

HIS: hospital information system; IT: information technology.

## Competing interests

The author(s) declare that they have no competing interests.

## Authors' contributions

MD designed research, analyzed data and wrote the manuscript. ME designed HIS reports. HB designed research and reviewed the manuscript.

## Pre-publication history

The pre-publication history for this paper can be accessed here:


